# Data Decomposition Techniques with Multi-Scale Permutation Entropy Calculations for Bearing Fault Diagnosis

**DOI:** 10.3390/s18041278

**Published:** 2018-04-21

**Authors:** Muhammad Naveed Yasir, Bong-Hwan Koh

**Affiliations:** Department of Mechanical, Robotics and Energy Engineering, Dongguk University-Seoul, 30 Pildong-ro 1 gil, Jung-gu, Seoul 04620, Korea; naveed.yasir@gmail.com

**Keywords:** rolling element bearing (REB), fault detection and diagnosis (FDD), local mean decomposition (LMD), multi-scale entropy (MSE), sample entropy, permutation entropy (PE), multi-scale permutation entropy (MPE)

## Abstract

This paper presents the local mean decomposition (LMD) integrated with multi-scale permutation entropy (MPE), also known as LMD-MPE, to investigate the rolling element bearing (REB) fault diagnosis from measured vibration signals. First, the LMD decomposed the vibration data or acceleration measurement into separate product functions that are composed of both amplitude and frequency modulation. MPE then calculated the statistical permutation entropy from the product functions to extract the nonlinear features to assess and classify the condition of the healthy and damaged REB system. The comparative experimental results of the conventional LMD-based multi-scale entropy and MPE were presented to verify the authenticity of the proposed technique. The study found that LMD-MPE’s integrated approach provides reliable, damage-sensitive features when analyzing the bearing condition. The results of REB experimental datasets show that the proposed approach yields more vigorous outcomes than existing methods.

## 1. Introduction

Mechanical defects occur in rolling element bearing (REB) due to wear, fatigue, corrosion, overload, and misalignment. To guarantee the safety of a system exposed to harsh environments, reliable condition monitoring strategies are required. Monitoring and evaluating the current condition of the REB is significant to many researchers for ensuring high operational efficiency. The temperature, vibrational response, and lubrication state are few features of non-destructive condition monitoring. Since the REB system requires a continuous operational state for an indeterminate period, vibration response data can be easily obtained from transducers mounted in the vicinity of the system. Bearing fault detection and diagnosis (FDD) problems have been popular research topics for many decades [1,2]. A variety of techniques exploit vibrational data to assess damage-sensitive features for diagnosis, and many methods use feature extraction and classification tools to monitor the state of the system’s components based on statistical deviation from normal operations [3,4].

Some of the signal-based techniques explored in data analysis processes, such as frequency-spectrum, time-statistical, and time-frequency analysis, provide simultaneous information on the time and frequency of the system response [3]. The time-frequency approach has been employed in different ways for REB fault detection. Defects typically occur in the REB due to micro-cracks and abrasions resulting in strong nonlinear, non-Gaussian, and nonstationary profiles in its vibrational response. Several time-frequency techniques have their own lacunae. Windowed Fourier transform (WFT) [5] has a fixed window; Wigner Ville distribution (WVD) [6] causes cross-term interference when dealing with multi-component signals; short-time Fourier transform (STFT) [7] has a fixed analysis window; and Wavelet transform (WT) has different wavelets that should be predefined for each component [8,9]. Wavelet analysis may be applied to FDD problems in rotating machines that can decompose the vibrational signal only if a rectangular time–frequency partition is available, a requirement that would fail to guarantee the instantaneous frequency of each resulting component obtained by WT. Power spectrum analysis and fast Fourier transform (FFT) has been employed to decompose the original signal into its component form for REB faults detection [10].

The time-domain technique exploiting statistical features such as kurtosis, entropy, RMS, skewness and impulse factor are used as benchmark parameters to assess the bearing conditions. The computational intelligence techniques such as machine learning and support vector machine (SVM) have been used to classify the characteristics of the regular healthy and damaged systems [11,12,13]. Moreover, some of the fault detection problems have been solved by using fuzzy-based techniques [14,15]. Empirical mode decomposition (EMD) has also been an effective tool that provides an advantage over other signal analysis techniques because it tackles non-stationary and non-linear vibrational signals [16,17]. The EMD is inherently combined with the concept of Hilbert transform (HT) to obtain frequency modulation [17]. EMD decomposes the multi-component signal into an amplitude and frequency modulated (AM-FM) signal that produces several intrinsic mode functions (IMFs). In each IMF, HT can calculate the corresponding instantaneous frequency (IF) and instantaneous amplitude (IA). However, there are some deficiencies in EMD, such as mode mixing, envelope line, and end effect, which are still under investigation in recent literature. Furthermore, the impulsive negative instantaneous frequency can appear when computing the instantaneous frequency using the Hilbert transform.

A self-adaptive time–frequency analyses technique called Local Mean Decomposition (LMD) was introduced and used to analyze electroencephalogram (EEG) signals [18]. The LMD generates several product functions, each of which is composed of an AM-FM signal. Naturally, this multi-component time–frequency signal can be reconstructed in its original form by summing up all of the instantaneous amplitudes and frequencies of each product function. Hence, the LMD is suitable for analyzing non-stationary signals as well as extracting fault-sensitive signatures from the REB response. Additionally, multi-scale entropy (MSE) was proposed to address the drawbacks found in samples that handle long-term data configuration [19]. The MSE is widely used in a range of data processing applications, such as the analysis of biological signals [20,21], postural control [22], and the vibration of REB [23].

Here, the ingenuity of the MSE is that it is considered a two-step movement: (1) a coarse-grained time series is used to develop illustrations of the system dynamics at variant time scales; and (2) a sample entropy is used to measure the regularity in the time series data [19]. The integrated approach of LMD and MSE has already been introduced in fault detection of REB by Liu et al. [23]. It has also been reported that a single scale method, such as sample entropy or approximate entropy, is unable to provide transparent pictorial information on bearing conditions [13]. Among others, permutation entropy (PE) has recently been used as a damage-sensitive feature after performing the decomposition of its vibrational signal [24].The concept of multi-scale permutation entropy (MPE) is based on the coarse-grained process and calculating the PE at each scale factor. It has been established and implemented for signal processing of human brain activity in applications such as EEG analysis [25], electrocardiogram (ECG) analysis, [26] and REB monitoring [27].

In the present work, a recently developed concept of MPE integrated with LMD has been applied to monitor the condition of an REB system. The obtained time-series data collected from the rotating systems (bearings or gears) have been convoluted and contain the data structure of multiple dimensions. Here, the algorithm of LMD-MPE is projected, and its application to REB shows an enhanced ability to classify the condition of the REB. This paper consists of five sections. In Section 2, the Shannon entropy, sample entropy, PE, MSE, and MPE are explained in detail. The aforementioned methodology is implemented on experimental data and the validity of the proposed method is described in Section 3. In addition, the result of ANOVA variance test [28] and feature classification are performed to discuss cross-verified results. Finally, a concluding statement and future research direction are presented.

## 2. Preliminaries

**Figure 1:**
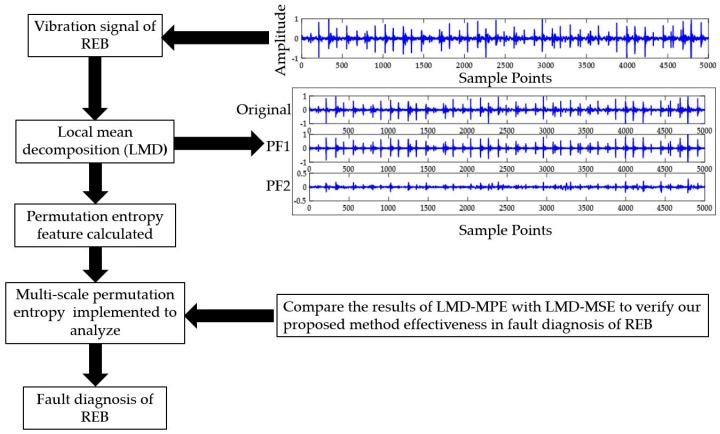
The proposed methodology (LMD-MPE) scheme for REB fault diagnosis.

The overall schematic of the implemented methodology of this study is depicted in Figure 1. First, the vibrational signal was decomposed by LMD [18] and the sample entropy and PE were then calculated as damage-sensitive features. The details of the methodology are discussed in the following subsections.

### 2.1. Principle of LMD

The LMD method is composed of envelope and frequency modulation of the decomposed form of multicomponent signals. The primary aim of the time–frequency LMD method is to decompose the multi-component AM-FM signal into mono-component signals using the local mean values of the signals and envelope components. The LMD is a composite of the product functions that consist of frequency and amplitude modulation of the signal. The frequency modulation incorporates meaningful information regarding the vibrational signals and produces amplitude modulation. The product of the envelope-modulated signal and modulated frequency are called the product function [18]. The summarized formula is given as

(1) $$x(t)=\sum_{i=1}^{j}PF_{i}(t)+u_{j}(t)$$

where PF is the product function and *u* is the residue, which becomes monotonic or a constant value at the end of the process (details of the LMD method can be found in References [18,23]). This is a suitable reason for LMD to be considered appropriate for non-linear and non-stationary signals in rotatory signals. Secondly, LMD is a self-adaptive method despite any fixed windowing or the presence of another fixed parameter, so it can cater to the sudden change in the signal. The other method combined the LMD method and MSE to keep the problem solution-efficient.

### 2.2. Principle of Entropy

Entropy is a parameter that assesses the level of disorder in the data. In other words, it represents a lack of information in the signal or data. The basic concept of entropy was first developed by Shannon and thus named the Shannon entropy [29]. Pincus brought forward the methodology of approximate entropy as a measurement tool for a complex system [30]. Richman et al. first introduced sample entropy and compared approximate entropy in the analysis of physiological time-series data [31]. Approximate entropy is implemented for noisy, short, real-time data to predict chaotic oscillation change and sometimes produces an inconsistent result because its performance highly depends on the size of the time-series data. Unlike approximate entropy, sample entropy is not dependent on the length of time-series data. It is based on the calculation of a negative natural logarithm of a distance between two vectors. The ones are maximum norm values where self-matching data points are excluded. This mathematical expression can be written as:

(2) $$H(x)=-\sum p(x_{i})\mathrm{logp}(x_{i})$$

where p(*x_i_*) represents the density function of unsystematic components in data points and the logarithm is based on two natural functions.

The PE can be used to assess chronological information embedded in time-series data. It is simple in computation and its results are consistent. However, the PE of the *m* dimensions [13] always needs to be appropriately selected to achieve the best result. The more substantial value of *m* is costly with respect to computational run-time. Given the time series x with delay factor *τ* and embedded dimension *m*, the delay vector can be given as [13]:

(3) $$x_{i}^{m}=[x(i),x(i+\tau ),\ldots ,x(i+(m-1))\tau ]$$

Here, the time delay factor is defined as *τ* and *m* is an embedded dimension. Each permutation of π and the relative frequency can be obtained as [13]:

(4) $$p(\pi )=\frac{Number\{t|t\leq T-(m-1)\tau ,x_{t}^{m}\;has\;type\;\pi \}}{N-(m-1)\tau }$$

The PE of the above time series in Equation (3) of *m* dimension is defined in the arrangement of the Shannon entropy. It can be written as:

(5) $$H_{\mathrm{PE}}(m)=-\sum_{i=1}^{m!}p(\pi_{i})\ln(p(\pi_{i}))$$

The normalized PE (NPE) can be described as:

(6) $$H_{\mathrm{NPE}}(m)=\frac{H_{\mathrm{PE}}(m)}{\ln(m!)}$$

As before, the PE value depends on the embedded dimensions *m* and delay factor *τ.* If *m* decreases, the algorithm will not work correctly for the detection of anomalies that occur in the time-series data. Specifically, a lower value of *m* will result in a lower value of PE, and it becomes hard to detect anomalies in different defect conditions. On the contrary, the high value of PE allows for the relatively easy identification of anomalies in regular and irregular time-series data. For calculation, we used the Shannon entropy H_PE_ of Equation (5).

### 2.3. MPE and MSE

Costa first introduced the MSE technique to analyze time-series data [19,21] based on the concept of sample entropy. PE is another entropy-based algorithm, but it has a single value. Thus, PE can be integrated with the multi-scale procedure to form a new entropy-based feature called MPE [13]. Both MSE and MPE employ a coarse-grained procedure that yields new time series data by using the mean value in each non-overlapping segment of the equal length of the output variable. Suppose that we have *N* data points that represent the time series; coarse-grained data points can be calculated using Equation (7).

(7) $$y_{j}^{\left(\tau \right)}=\frac{1}{\tau }\sum_{i=(j-1)\tau +1}^{j\tau }x_{i},1\leq j\leq \frac{N}{\tau }$$

**Figure 2:**
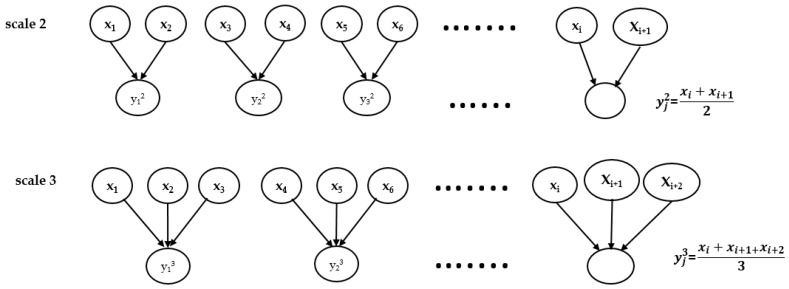
The schematic diagram of a coarse-grained procedure for scales two and three. Figure reproduced from Ref. [21].

Here, *τ* is the non-overlapping window length of the data point and averages the data points in the corresponding window. The MSE calculates the complexity of time-series data by calculating the sample entropy of a particular set of data. The coarse-grained technique gives the average of non-overlapping, consecutive data points. The sample entropy can be obtained through Equation (1) at each coarse-grained scale factor in the case of MSE and MPE. The MSE technique was initially designed to measure the change correlated with the data series on multiple time scales. This technique provides more information in the case of a single-value scale. The schematic diagram of the coarse-grained process is depicted in Figure 2 [21]. Both MSE and MPE methods use the coarse-grained technique prior to calculating the sample entropy or PE entropy.

## 3. Experimental Evaluation

### 3.1. Characteristic Properties of Bearing Data

The experimental data for this study was accessed from the bearing data center at Case Western Reserve University (CWRU), USA [32]. The REB model of 6205-2RSJEMSKF was used by CWRU and the time-series data were recorded through accelerometers mounted on the bearing housing with magnetic bases. The accelerometers were located at the motor-drive and fan-drive ends. In this study, we used the REB data from the motor-drive end. The speed of the motor-drive was 1797 rpm and varied with the change in loading. The sampling frequency was 12 kHz, and, thus, a total of 5000, 2048, and 1024 sampled data points were selected to form a set of segments as outlined in Table 1. The REB condition was delineated as being healthy normal or defective in the rolling element, inner-race, or outer-race. Furthermore, four different fault sizes, 0.007, 0.014, 0.021 and 0.028 inches, were created on the inner-race, outer-race, and rolling element. Table 1 also describes cases of REB bearing damage sorted by the lengths of data and severity of damage in different locations. Table 2 illustrates the detail of the inner-race fault size on the REB datasets used in Case 1.

The next stage involved the decomposition of the bearing signal using the time-frequency analysis LMD technique. First, the original signal was decomposed into several product functions. The sample entropy and PE values were then quantified from each product function to calculate the MSE and MPE. For verification purposes, the values from the proposed LMD-MPE technique were compared with those from previously published LMD-MSE technique results [23].

All three cases were obtained from the drive-end location. The motor speed was 1797 rpm and the motor load were zero horsepower.

### 3.2. LMD Implementation on the Data from REB Cases 1–3

In this section, we investigated three different damage sizes instigated on the inner-race of the REB in Case 1. The resulting shock impulses by defect were modulated with the normal vibrational signal of the REB, which made it a signal composed of different sources, such as the original and shock-induced impact signals. The vibration signal from REB was decomposed into multiple components and a monotonic residue for feature extraction through LMD. The sampling frequency was 12 kHz and the rotational frequency of the inner-race was 40 Hz. The calculated rotational frequency of REB with respect to the shaft is 85.7 Hz.

**Figure 3:**
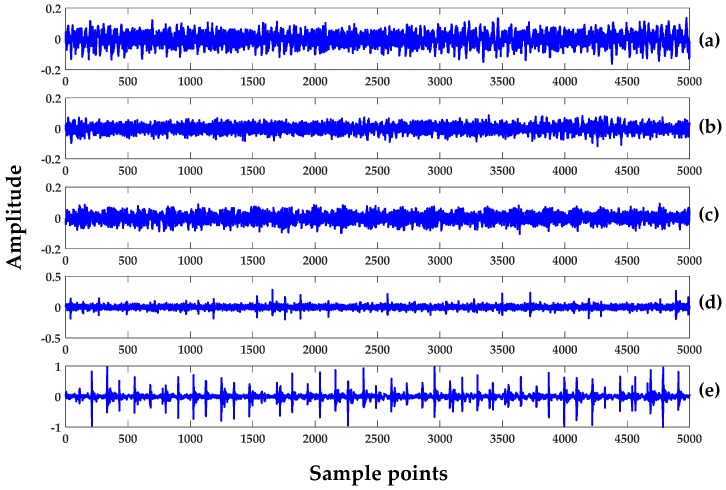
Time-series of REB for Case 1 of same segment set: (**a**) Healthy set 1 (H1); (**b**) Healthy set 2 (H2); (**c**) Damage set 1 (D1); (**d**) Damage set 2 (D2); and (**e**) Damage set 3 (D3).

The time series for all of the datasets (see Table 2) in Case 1 are shown in Figure 3. The total length of the data is 600,000 points and they were further divided into several datasets for the convenience of computation. Each dataset consists of 5000, 2048, and 1024 points in Cases 1, 2, and 3, respectively, as described in Table 1. While eight data segments were used in Case 1, only five segments were used for Cases 2 and 3. Both H1 and H2 were the same types of healthy signals (or the baseline). The only difference was that H1 was the standard baseline signal for all signals acquired in the CWRU dataset at zero motor load (i.e., filename: Normal_0). The H2 was a particular case for a healthy signal and became a baseline for outer-race damage recorded in different instances.

The first eight segments of Case 1 were considered to show a general trend in damage-sensitive features when monitoring the bearing condition. The total segments consisted of 40,000 (8 × 5000) data points. The implementation of LMD on Case 1 that yielded product function was integrated with MSE in every health condition of REB to visualize the fault extraction investigation trend. A single damaged area created on the surface of an inner-race had three different severity conditions, as described in Table 2. Furthermore, two independent sets for the normal healthy condition were analyzed.

**Figure 4:**
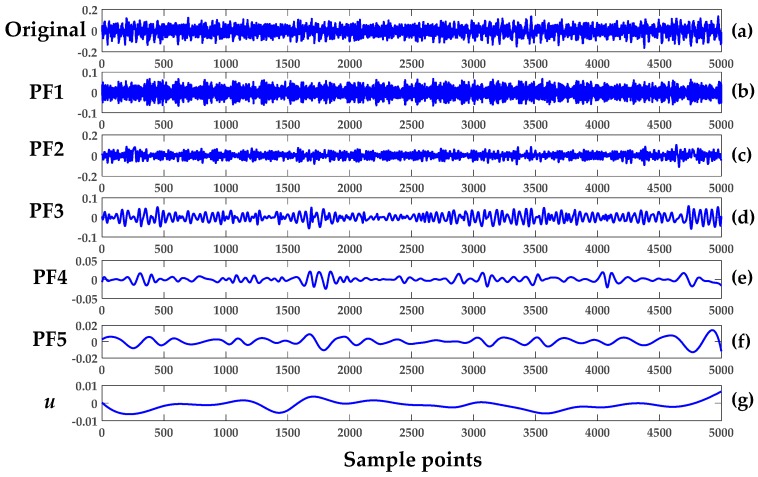
LMD of normal healthy Case 1: (**a**) original H1; (**b**) PF1; (**c**) PF2; (**d**) PF3; (**e**) PF4; (**f**) PF5; and (**g**) *u* is the residual signal.

**Figure 5:**
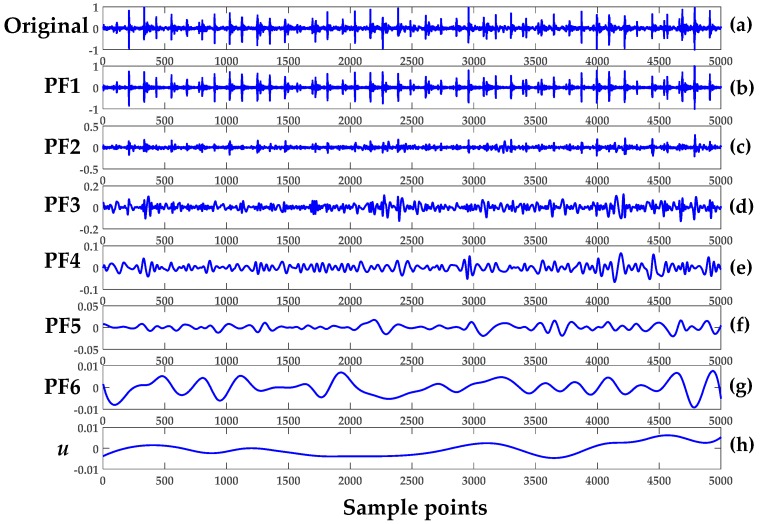
Product functions of the damage Case 1 set: (**a**) original (D3) vibrational signal; (**b**) PF1; (**c**) PF2; (**d**) PF3; (**e**) PF4; (**f**) PF5; (**g**) PF6; and (**h**) *u* is the residual signal.

The first five segments of the REB—healthy set 1 (H1), healthy set 2 (H2), damage set 1 (D1), damage set 2 (D2) and damage set 3 (D3)—were analyzed to verify the LMD methodology. The five product functions and a residual signal generated after LMD implementation are shown in Figure 4. The first segment of H1 of Case 1 was used. Next, the multiple components of segment 1 of D3 was then decomposed into six product-functions plus one residue, as illustrated in Figure 5. Each plot provided a total of 5000 data points. It should be noted that number of product functions depended on the complexity of the signal, and the higher the signal complexity, the higher the number of product functions.

Apparently, the first few product functions inherited most of the features from the original vibrational signal. In Case 1, the first two product functions (PF1 and PF2) were used to demonstrate feature extraction from the original healthy signal. The basic concept of LMD is to remove the higher frequency with an iterative algorithm and obtain the mean values of all of the new signals. The new envelope signal is subtracted from the original one and the subtracted signal becomes the next input signal. In the end, the decomposed signal is then formed into a monotonic signal, as is clearly visualized by Figure 4g and Figure 5h.

### 3.3. MSE and MPE Implementation on Experimental Data

First introduced by Costa [17], MSE was developed to estimate the degree of complexity of time-series data over multiple time scales. Sample entropy is the most common tool used in MSE to check regularity in data. It is observed that the coarse-grained data sample entropy value reduced in accordance with the increase in the time scale factor. The MSE and MPE exhibited differences in extracting defect features with different degrees of severity. The values of MSE and MPE vary with different product functions, time scales, and damage conditions. Therefore, MSE and MPE can be effectively employed as a parametric scale to assess the abnormality in measurements of the signal, such as kurtosis and entropy.

#### 3.3.1. The Integrated Approach of LMD and MSE for Cases 1–3

**Figure 6:**
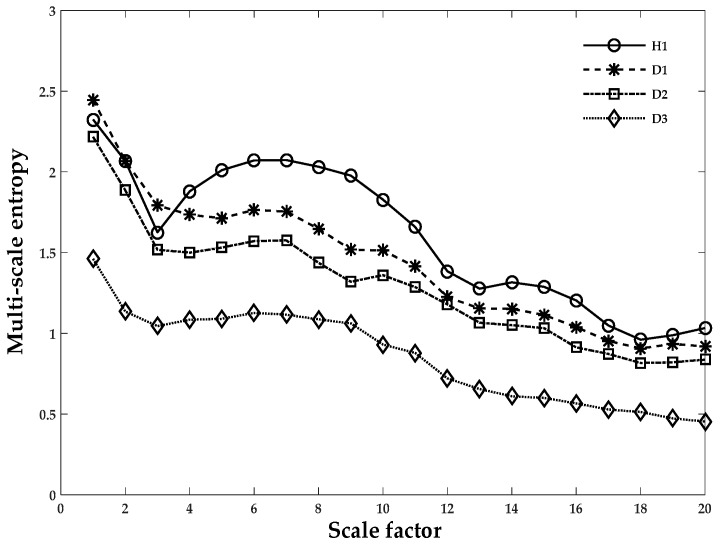
The original REB signal for Case 1. An average of eight segments were in each set. Scale factor 20 vs. multi-scale values (sample entropy) were used to obtain the results of the MSE technique.

**Figure 7:**
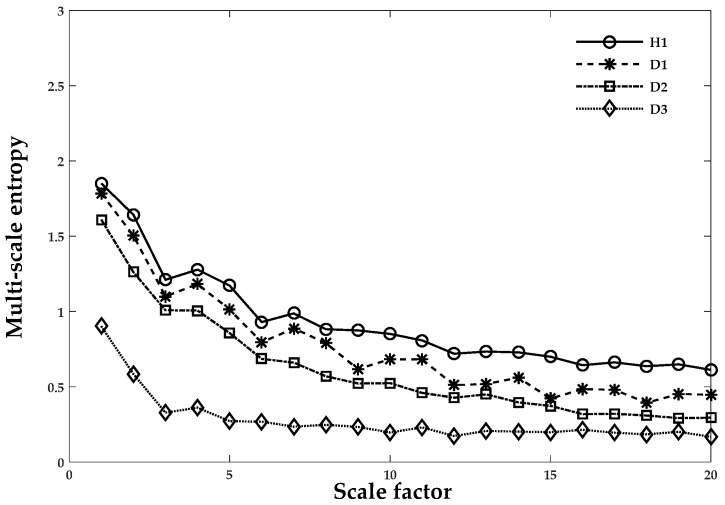
REB Case 1 and Product Function 1 with an average of eight segments per set. Scale factor 20 vs. multi-scale values (sample entropy) were used to obtain the results of the MSE technique.

Here, MSE was regarded as an index and a feature for characterizing the complexity of the chaotic time series data of REB. In the next stage of fault extraction, the combined LMD and MSE approach was used to monitor the condition of an operating REB. The overall process went through the following steps:

1. In Cases 1, 2, and 3, the vibrational signal was sampled as shown in Table 1. To establish succinct benchmark results, eight, five, and five sets of segments were used in Cases 1–3, respectively.
2. The LMD technique in Equation (1) was used to obtain the product function and residual signal of the damaged REB conditions. The first five product functions were selected to extract meaningful information. Figure 4 and Figure 5 show the LMD implementation of the data for Case 1. For MSE, the scale factor was a positive integer, and for Cases 1–3, it was selected as 20, 15 and 15, respectively.
3. A graph of the LMD-MSE result was constructed with the help of original REB signal and Product Function 1 of all defect conditions of Case 1. The output of the MSE was shown in Figure 6 and Figure 7 for original signal and Product Function 1, respectively.
4. A proper classifier for a damage-sensitive feature can be used to make the result clearly distinctive from the different REB damage conditions.
5. In Figure 6 and Figure 7, distinctive MSE points for different damage cases cannot be easily separated, especially in the case of H1 and D1. The other factor, which did not allow for distinction, may be considered a measurement noise.
6. The comparison between the original and different faulty conditions showed that the data from the damaged REB was more separable in the MSE versus Product Function 1 at the coarse-grained time factor τ = 5 and may be a parameter for measurement factors such as kurtosis and entropy.

Figure 6 and Figure 7 show the MSE method applied to both healthy and different damage severities and the same damage type cases to illustrate the differences between them. Curves from the original vibrational signal and Product Function 1 after applying LMD are depicted in Figure 6 and Figure 7, respectively. In these figures, the data points are given as an average of the eight segments in each case scenario. In Figure 6, when the scale factor was less than five, the MSE curve of all health condition cases exhibited ambiguity in the sample entropy values and had the same values or a higher value of D1 than H1. The few instances where the values of D1 and D2 were not clearly discernable were at 13 and 15, as shown in Figure 7. After decomposition through LMD techniques, the three different damage severities (D1, D2, and D3) could be easily distinguished from the normal healthy (H1) data, as illustrated in Figure 7. At this stage, we discussed the conventional techniques and demonstrated that the proposed method or LMD-MPE properly performs with relatively small computational time and has a more robust result. In the next section, we will establish a comparison between Case 1 and Case 3 for PE calculation of the validity of the REB data. This comparison is based on two criteria: (i) the same damage type but different damage intensity, such as 0.007, 0.014, and 0.021 inches (Case 1); or (ii) the same intensity of damage but different damage types, such as the 0.021 inches damage intensity (inner race, outer race, or rolling element, similar to Cases 2 and 3). In the next section, since Cases 1–3 are scenarios based on different damage severities and data lengths, we developed a reliable solution in which whatever scenario was presented, the PE calculation worked successfully.

#### 3.3.2. PE for Cases 1–3

**Figure 8:**
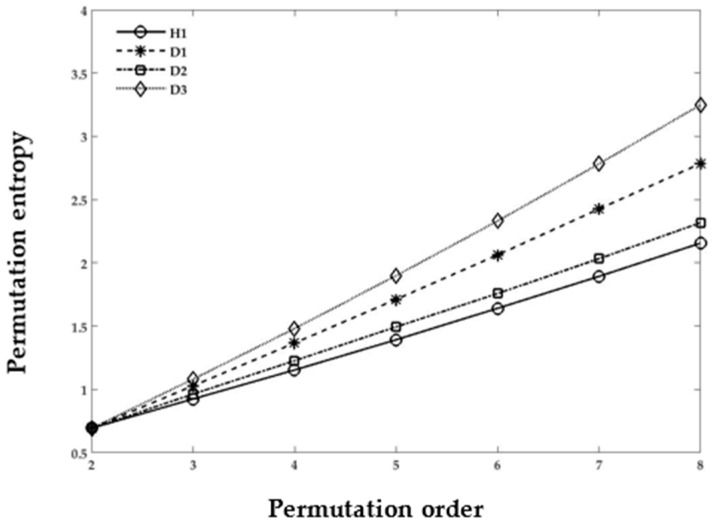
Product Function of Case 1, damage size: 0.007, 0.014, and 0.021 inches; damage type: outer-race; data points: 1024; PE calculation with different *m* permutation order.

**Figure 9:**
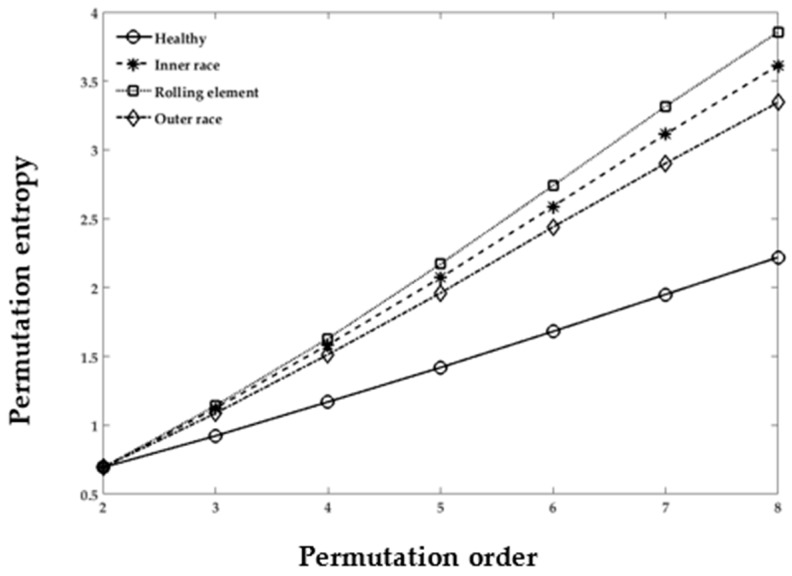
Product Function of Case 3, damage size: 0.021 inches; different damage locations; PE calculation with different *m* permutation order.

PE was originally proposed to examine the complexity of the natural time series data [33]. PE offers several advantages, such as less computational complexity and stability in the results, over other entropy calculation techniques. The calculation of PE values depends on the *τ* delay factor and *m* dimensions. In Figure 8 and Figure 9, the embedded dimension *m* varies from 2 to 8 in obtaining the optimal value of the permutation order. All the healthy condition sets of Cases 1–3 have variations in the PE value. The theory also states that the smallest value of the embedded dimension *m* produces inconsistent results. However, for the higher value of *m*, the PE yields remarkably independent metrics that can be used to distinguish the healthy and damaged REB datasets, as depicted in Figure 8 and Figure 9. The figures also show the implementation of PE in the decomposed REB vibrational signals. All the REB conditions manifest recognizable separation from each other in the healthy and damaged cases (i.e., inner, outer race, and rolling element), as shown in Figure 8 and Figure 9. Note that the “H1” and “Healthy” of both graphs have approximately equal values. The case of “Outer race” in Figure 9 might be compared with D3 in Figure 8. Moreover, it has been shown that the values are approximately equal in the graphs. The results show that, when we have either different data sizes or different damage types, the PE values can distinguish the differences among REB conditions (i.e., healthy, inner-race, outer-race, and rolling element) by changing the value of *m* in the permutation order.

#### 3.3.3. MPE for Cases 2 and 3

**Figure 10:**
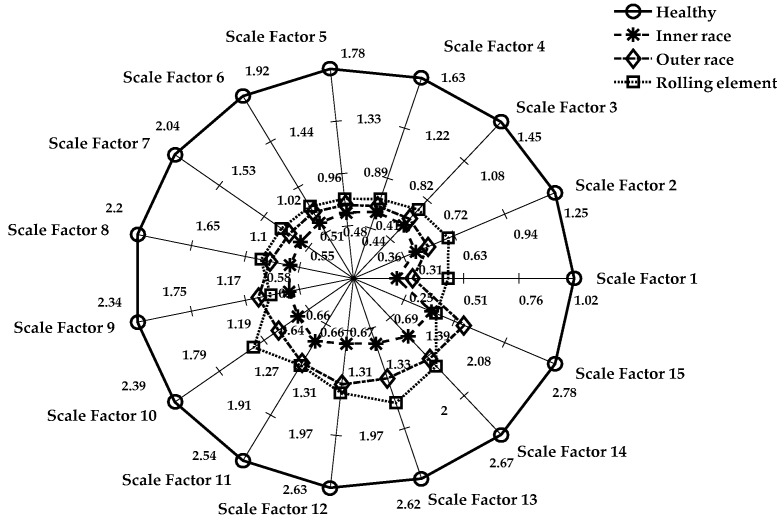
Spider plot for MSE of the PF4 (Case 2): damage size is 0.021 inches.

**Figure 11:**
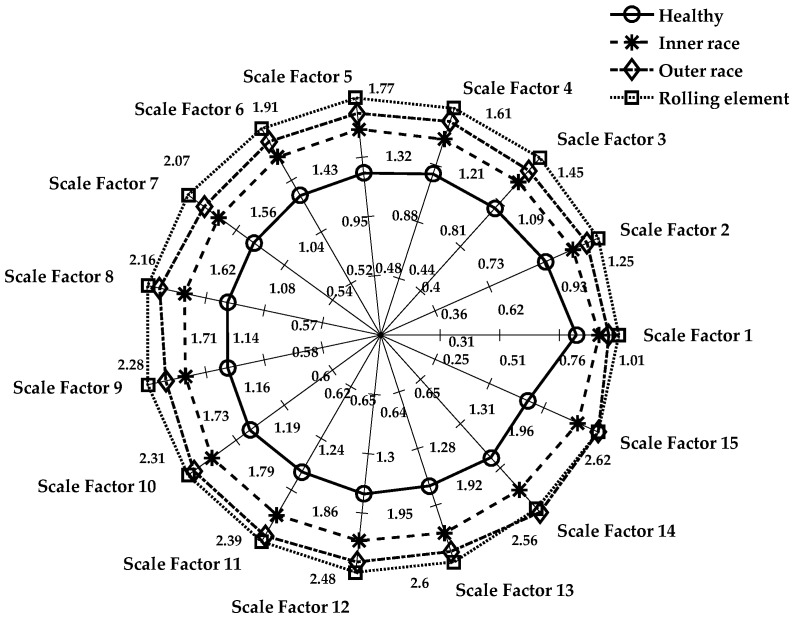
Spider plot for MPE of PF4 (Case 2): damage size is 0.021 inches.

The MPE algorithm was implemented in the REB data, and the preprocessing for the decomposition of the signal remained the same as in the LMD method. The length of data samples *N*, delay factor *τ*, and embedding dimension *m* are effective parameters in the calculation of MPE. If *m* is a small value, then few characteristics will emerge that will produce a few dynamic changes, and the changes that happen in the time series cannot be determined precisely. On the other hand, if the value of *m* increases, then the segment space of the time series will be standardized, which requires significant computing time and can be interpreted inaccurately. Therefore, the selection range of *m* is 4–7 in general [13,27]. We chose *m* = 4 and the delay factor *τ* = 1 to calculate PE in a later context. In the next stage, the integrated approach of LMD and MPE was used to detect the features of damaged REB. Again, the MPE measured the complexity of the signal at a different scale factor and different severity of bearing damage. The whole process included the following steps for both the MSE and MPE methods:

1. Only five segment sets of each health condition were investigated among the bearing data of Cases 1–3, as shown in Table 1 (i.e., five segments of all cases and their subclasses, such as healthy, inner race defect, outer race fault, and rolling element defect, were investigated.)
2. The LMD method was used to calculate the product function of each damaged condition. The first five product functions were selected to obtain meaningful information regarding the bearing condition. Figure 4 and Figure 5 show the LMD implementation from Equation (1).
3. The scale factor was an integer number and selected as 15 for both Cases 2 and 3. The coarse-grained calculation was performed through Equation (7).
4. The PE values were calculated for each coarse-grained signal using Equation (5). The sample entropy values were calculated for the MSE graph.
5. Then, the MSE and MPE graph was created with every cross-grained point. The respective sample entropy and PE values are depicted in Figure 10 and Figure 11.
6. Figure 10 shows a spider plot of MSE preprocessed by LMD of the original REB signal. The same original dataset of REB was used to produce Figure 11 after performing the LMD-MPE algorithm. The different damage location of the same damage severity could not be identified precisely, as shown in Figure 10. The same original REB condition scenario dataset after processing using the LMD-MPE approach presented in Figure 11 shows a clear separation among the damage locations of the same damage severity.

The spider plot is a graphical representation of multivariate data projected onto a two-dimensional graph of more than three quantifiable variables. These variables represent multiple axes, which all start from a single central point. Here, the spider plot is used to demonstrate the comparison of healthy condition cases of REB (i.e., healthy, inner race, outer race, and rolling element) at different scale factors. It also explains the differences among the values of each healthy condition at separate axes of the scale factor. The value of the scale factor starts from the center point (origin) and ends at the outer circle ring. In Figure 10, the healthy case has a maximum value, which shows the outer circle of the spider plot. All the faulty conditions (inner-race, outer-race, and rolling element) are shown toward the center of the circle. In a few instances, the outer race sample entropy value is higher than the rolling element sample entropy value (i.e., at scale factors 9, 11 and 15). In Figure 11, defects on the rolling element and inner-race have the same entropy value only at scale factor 15, which shows the robustness of MPE. Note that the MSE and MPE results have the same damage size and product function, and the entropy values represented are the averages of five segment sets. The same damage severity of different damage locations shows that the bearing defects are more easily separable in the MPE than MSE toward the same original REB signals, as shown in Figure 11.

It is shown that the healthy and defective REB signals can be accurately discerned, but it becomes very hard to identify the types or locations of the fault occurrences in Figure 10. Moreover, Figure 11 shows the LMD-MPE method using a spider plot, which can easily identify all health condition cases of REB. Here, in this spider plot, the healthy case signal has been positioned near the center of the circle. All damage cases (inner-race, outer-race, and rolling element) can be identified clearly except the outer-race and rolling element at scale factor 15. The MSE and MPE algorithms were applied to the same PF4 signal, and the results are shown in Figure 10 and Figure 11. The scale factor is a significant parameter of the MSE and MPE that was clearly illustrated using a spider plot. Figure 10 and Figure 11 show the entropy values corresponding to every scale factor from 1 to 15. The function mapped with a total 15 scale factors and MPE shows separable entropy values compared with that of the MSE case, as shown in these figures.

#### 3.3.4. Comparison of MSE and MPE Results of Case 2 Using Box Plot

**Figure 12:**
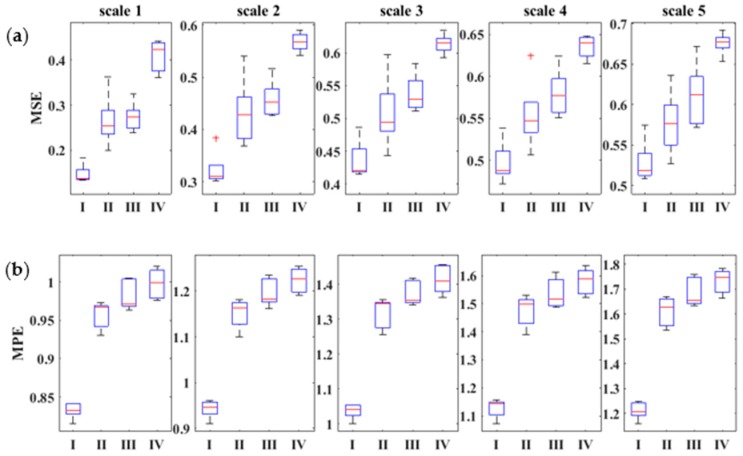
Boxplot for the MSE and MPE of the first five scale factors: (**a**) MSE (multi-scale entropy); and (**b**) MPE, where I is a healthy signal, II is inner race, III is outer race, and IV is rolling element.

To validate and compare the proposed method, a boxplot of five segments is presented in Figure 12. The first five-scale-factors standardized boxplot indicates the distribution of data by minimum, maximum, median, first, and third quartile values, as shown in Figure 12. The separation between the median of the normal healthy and damage cases, particularly at all mentioned scale factors 1–5, is remarkable. The calculated value of the median of the different defect condition cases shows their differences. The size of the interquartile range (IQR/variation box) of MPE (Figure 12b) is more compact than that of the MSE (Figure 12a), which shows that the LMD-MPE method has more certainty in the PE value calculation than the sample entropy calculation method, LMD-MSE. The whisker of the scale factor of the MSE technique is longer than the MPE shown in scale factors 2–5. The data outlier (positive red mark) in scales 2 and 4 (shown in the LMD-MSE technique) shows that some inconsistent values appear during the calculation of the sample entropy. However, in the LMD-MPE technique the outlier did not exist in all scale factors, as shown in Figure 12b. These points validated that the LMD-MPE technique shows superior distinction between the damage conditions of REB compared with the prior technique. The LMD-MPE technique offers a new prospective method for tackling the REB fault detection problem.

## 4. Discussion

The ANOVA statistical test [28,34] was also implemented in each scale factor case to verify that all the healthy conditions are expressively diversified among each healthy condition of REB (i.e., healthy, inner-race, outer-race, and rolling element defects). The ANOVA test was also employed to determine which method (LMD-MSE or LMD-MPE) provides better performance in the bearing monitoring problem. In the results of the ANOVA tests, the sum of squares between the two groups had lower MSE values (see Table 3) than MPE (see Table 4). The *p*-values were less than 0.05 in both the LMD-MSE and LMD-MPE techniques, which showed significant results at P. The F-test value was the ratio of variation between the group means to the variance within the damage condition groups means. The low F-test value shows that the group means were close to each other, and the high F-test shows a condition where the variability of the group means was large relative to the group’s variability. This means that the F-test could be a measure of separation among damage group means, as shown in Table 4. It is shown that, in both scale factor cases, the F-test (LMD-MSE = 27.49, LMD-MPE = 102.55) were higher than F-crit (3.24), as shown in Table 3 and Table 4, respectively. These values indicate that the four REB health conditions comprised of the healthy signal, inner race, outer race, and rolling elements were statistically and significantly different from one another. However, the F-test of LMD-MPE (102.55) was much higher than the F-test score of LMD-MSE (27.49). Therefore, it can be concluded that the LMD-MPE method is better than LMD-MSE. Moreover, in the datasets of LMD-MSE, all values were very close to the mean, resulting in a small variance (SS = 0.15) between the four REB healthy conditions. However, the datasets of the LMD-MPE values were spread further away from the mean, leading to a more substantial variance (SS = 0.25) between the four signal features. The total variance of MPE was 0.257, which was higher than that of the MSE (0.179).

Additionally, classification has been performed using the features from LMD-MSE and LMD-MPE. For comparison, MATLAB [35] has been employed for classification of over 15 datasets of REB Case 2. Here, the time delay factor and embedding dimension is set to unity and four, respectively. The label of healthy and damage condition is applied as an input for supervised learning and five-folds cross-validation are implemented for the training and testing of classifiers. Table 5 shows the result for average classification accuracy of various classifiers and area under the curve (AUC) of the receiver operator characteristics (ROC). The case of LMD-MPE provides relatively better accuracy in classification compared to LMD-MSE using all three classifiers. Considering the sensitivity and specificity, the classification result becomes successful as the AUC approaches to unity. Note that the result from the method of LMD-MPE gives more consistent value of AUC close to 1.00.

## 5. Conclusions

This research introduced LMD, a self-adaptive technique that can be integrated with MPE to tackle the problem of REB fault diagnosis using the test data from CWRU. The results of the proposed LMD-MPE method were compared with that of the previously used LMD-MSE technique. We found that LMD-MPE showed improvement and robustness in detecting an identical damage size (0.021 inches) with different damage locations (i.e., inner-race, rolling element, and outer-race) of REB compared with LMD-MSE. Specifically, the performances of LMD-MSE and LMD-MPE were reviewed and discussed through the graphical representation of spider plots. The results were also verified by two statistical analysis tests: boxplot and ANOVA. The boxplot showed that the LMD-MPE variation performance of the REB condition was higher than that of the LMD-MSE method. The ANOVA test also demonstrated that the variance between the groups was 0.25 in the LMD-MPE case, which can be compared with 0.15 for the LMD-MSE case. Moreover, the LMD-MPE and LMD-MSE results were compared using the SVM and KNN classifiers. The classifier accuracy and AUC values for LMD-MPE showed an acceptable performance in fault classification of REB. This anticipated technique can be readily extended to incorporate an online monitoring system for REB diagnosis. Future work can be directed toward fully self-automated or self-adaptive methods for fault diagnosis by calculating the MPE and optimizing the dimensions of PE for further development of a robust early-warning system in REB fault diagnosis using noisy and complex gearbox data in practice.

## Figures and Tables

**Table 1:** REB data cases based on different data lengths and damage types.

Case	Healthy	Damage Location	Fault Size (Inches)	Data Length (Samples)	Method Implemented
Case 1	Two healthy sets	Outer race	0.007, 0.014, 0.021	5000	LMD, MSE
Case 2	One healthy set	Inner race Outer-race Rolling element	0.014, 0.021, 0.028	2048	LMD MSE PE MPE
Case 3	One healthy set	Inner race Outer race Rolling element	0.014, 0.021	1024	LMD MSE PE MPE

**Table 2:** REB conditions for Case 1.

Set	Damage Condition
Healthy set 1 (H1)	Healthy REB
Healthy set 2 (H2)	Healthy REB
Damage set 1 (D1)	0.007-inch outer-race
Damage set 2 (D2)	0.014-inch outer-race
Damage set 3 (D3)	0.021-inch outer-race

**Table 3:** One-way ANOVA test for sample entropy at scale 2 of MSE.

Source of Variation	Sum of Squares (SS)	Degree of Freedom (DF)	Mean Square (MS)	*p*-Value	F-Test	F-Crit
Between Groups	0.1503	3	0.0501	1.5 × 10^−6^	27.4866	3.2389
Within Groups	0.0292	16	0.0018			
Total	0.1795	19				

**Table 4:** One-way ANOVA test for PE at scale 2 of MPE.

Source of Variation	Sum of Squares (SS)	Degree of Freedom (DF)	Mean Square (MS)	*p*-Value	F-Test	F-Crit
Between Groups	0.2446	3	0.0816	1.16 × 10^−10^	102.5448	3.2389
Within Groups	0.0127	16	0.0008			
Total	0.2574	19				

**Table 5:** Comparison of average of classifiers on REB data Case 2.

Classifier	LMD-MSE		LMD-MPE	
	Accuracy (%)	AUC	Accuracy (%)	AUC
Fine Gaussian SVM	66.3	0.94	87.1	1.00
Medium Gaussian SVM	88.0	1.00	90.4	0.99
Fine KNN	84.2	0.96	86.3	0.98
